# Design and implementation of efficient bootloader for endurance enhancement in flash memory storage systems

**DOI:** 10.1016/j.heliyon.2024.e26760

**Published:** 2024-02-28

**Authors:** Mehmet Ugur Kebir, Firat Kacar

**Affiliations:** Electrical-Electronics Engineering, Istanbul University-Cerrahpasa, Istanbul 34320, Turkey

**Keywords:** Flash memory, Endurance, Bootloader, Embedded system, Optimization

## Abstract

We propose a new bootloader design with dynamic boot addressing to increase the endurance of microcontroller flash memories and to use flash memory efficiently.

Although the final industrial products are not updated much, regular programming during the development and testing phase inefficiently reduces the endurance of flash memory. Especially after the pandemic, the problems experienced in the production/supply processes of products using semiconductor technology and the highly extended deadlines have inspired our work to extend the life of the products used in the development and testing phase.

Within the scope of the study, a bootloader design and implementation related to increasing the durability of flash memory, which affects the lifetime of microcontrollers, are explained.

When a new application installation request comes, it is aimed to provide the following without the need for any additional hardware; It is to ensure that the data to be written to the flash memory is shared homogeneously between sectors, to prevent unnecessary memory deletion, to shorten the time spent on installing applications, to increase memory endurance and to predict possible memory endurance problems.

## 1 Introduction

Debuggers are the tools used to load applications developed for embedded systems into the target system, test them, eliminate errors, observe the workflow and intervene when necessary [1]. Many modern microprocessors are designed to be compatible with debugger tools at the design stage [2]. Debug ports such as Joint Test Action Group (JTAG), Serial Wire Debug (SWD), In-System Programming (ISP), and Background Debug Mode (BDM) in final product development kits or microcontrollers are chip-level connection interfaces designed for debugger tools to work. Debug ports are usually hidden or disabled in order not to create security weaknesses in end-user products, except for specific purposes such as providing system verification in safety-critical projects or products that avoid high costs due to design changes [3]. Bootloader software is widely preferred since debugging ports are not often used in end-user products, the high cost of debugging tools, and the programming and updating needs of electronic firmware.

The primary purpose of bootloader software is to initialize hardware devices to write the application code to be loaded into memory without the need for external programmer hardware [4]. Flash memories are among the best options for storage in embedded systems, embedding user application programs, and bootloader software [5]. Although developers prefer Random Access Memory (RAM) for fast program loading in some cases such as test and experimental processes, flash memory areas are generally used to make applications permanent. Because flash memories can be electrically erased and programmed, there are limitations on retention time and memory durability [6].

Information about the retention time and memory durability of flash memories in microcontrollers is provided by the manufacturers [7]. There is also an inverse relationship between the number of Program/Erase (P/E) cycles performed in flash memory sectors and the time it will reliably hold data programmed in the sector [8]. Manufacturers typically specify the data retention period in flash memories assuming no erasing in the flash memory sector. As P/E operation increases in a flash memory sector, the amount of time that data can be reliably retained decreases [9].

Wear-leveling algorithms play a crucial role in flash memory systems. Their purpose is to distribute the P/E cycles evenly across the physical sectors, thereby extending the overall lifetime of the memory. By reducing the average number of P/E cycles in logical sectors, wear leveling helps to mitigate the risk of certain sectors reaching their endurance limit before others. This balanced distribution of P/E cycles ensures that data retention time in these sectors is prolonged, as the wear leveling algorithm prevents any individual sector from experiencing excessive wear. Consequently, wear leveling algorithms contribute to the overall longevity and reliability of the flash memory system by minimizing the likelihood of premature sector failure and data loss.

Frequent programming of development boards and microcontrollers during product development and testing phase reduces flash memory durability. Although the update frequency of industrial products after finalization does not seem to be a problem for flash memory durability, the problems in the supply chain that started with the pandemic [10][11][12][13] and the problems in semiconductor production have inspired us to work on increasing the life of flash memory in the development and testing activities used in the processes until the products are finalized.

Since the holding time capacity of flash memories used in microcontrollers does not interfere with memory integrity if programming is not done at intervals for many years, the focus of the study has shifted to the efficient use of memory durability. Therefore, all studies and applications within the scope of the thesis focused on memory durability of microcontrollers, ignoring memory retention times.

## 2 Background

### 2.1 Flash memory endurance

There are many advantages of using flash memory in microcontrollers. It has fast access speed and low heat dissipation, also it is noiseless [14]. However, flash memories have a limited endurance. Flash memory endurance can be defined as the erasing and programming lifetime of the memory. Unless the data written to an address of the flash memory that used in the microcontrollers is deleted, the same address cannot be overwritten again [15]. Erasing the flash memory can be achieved through two distinct methods: bulk erasure, where the entire memory is erased at once, or sector-by-sector erasure, where each individual sector is erased separately. Writing to the flash memory, on the other hand, does not have to be bulk or sectoral due to the microelectronic design of the memory. This situation reveals the fact that the write and erase cycles can take different times, and there are more case where the erase operation takes longer than the write operation. If we want to explain this situation with an example, let's assume that we load a program of 1 kilobyte size on a flash memory with 16 kilobyte sectors in a certain time. To reprogram the flash memory with a new program of 1 kilobyte size in a region within the previously programmed address range, we will first need to delete the 16 kilobyte memory sectors and then reload the new 1 kilobyte program. Erasing an entire sector to program a data block proportionally corresponding to 1/16th of the sector size increases both erase-write cycle times and reduces sector endurance. Besides, if a power failure occurs while erasing a 16 kilobyte flash memory sector, 16 kilobytes of data will be lost instead of 1 kilobyte.

Although the flash memories used by microcontroller manufacturers have varying sector sizes, the fundamental process remains unchanged: the entire sector is erased to make way for writing a program that is smaller than the sector size, starting from the sector's initial address. However, this approach carries significant disadvantages, such as the relatively slow erase cycles compared to the write process and the limited number of write/erase cycles that flash memories can endure. In addition to these drawbacks, microcontroller manufacturers have established the foundation of classical bootloader programs through the development platforms and software they provide to users. They have configured linker files, bootloader software, demo projects, and similar programs as examples to be loaded from a fixed or zero flash memory address.^1^^,^^2^ As a result, even if the size of the loaded program is considerably less than the sector capacity of the flash memory, since the next program loading will start from the zero or a fixed address of the flash memory, respectively erasing and writing operations will be performed in the same memory region. Considering the experiment and test stages in embedded software development processes, programming of microcontrollers with classical methods reveals a few points where flash memory management is quite inefficient. Especially in cases where the size of the program to be loaded is smaller than the flash memory sector is a very suitable subject for optimization. In the following topics, the mentioned disadvantages of flash memories will be discussed with the wear leveling algorithms and optimization studies without the need for additional hardware.

### 2.2 Classic bootloader designs

Bootloader designs are relatively small software in comparison to the memory size that can be programmed at the start of flash to function as an application loader as well as an update mechanism for applications running on the microcontroller. There are many different sizes and flavors to embedded boot-loaders. They can communicate over a variety of protocols such as Universal Synchronous and Asynchronous Receiver-Transmitter (USART), Controller Area Network (CAN), Inter-Integrated Circuit (I2C), Ethernet, Universal Serial Bus (USB) and the list goes on for as many protocols that exist [16]. Since the hardware configurations of each microcontroller family may differ, there is no standard bootloader software for all microcontrollers. Therefore, bootloader is needed for each type of the microcontroller according to their hardware features [17]. But in terms of functionality it is possible to describe a general bootloader mechanism.

**Figure 1:**
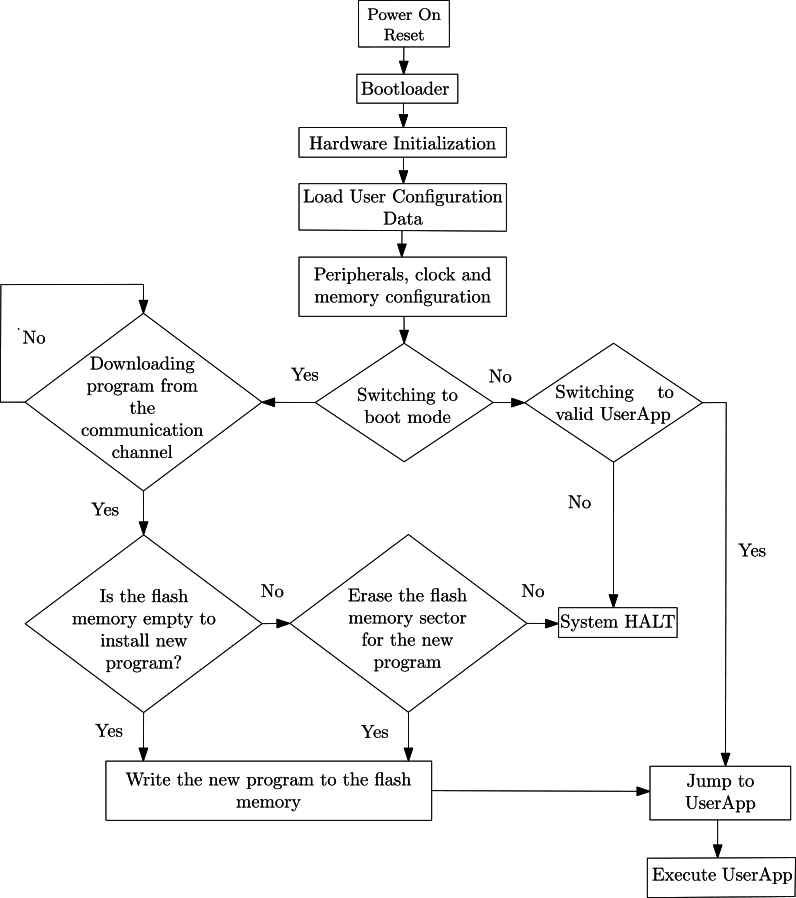
General Flow Diagram Of Classic Bootloader Design.

Since the bootloader software, linker file examples and demo applications offered to users by microcontroller manufacturers, start from the zero or fixed address of the flash memory in the programming process, the classic bootloader definition has been made. Classical Bootloader (CB) software can come in various sizes and different features, but in general, the operation of a classic bootloader software is relatively standard [16]. The general flow diagram of classical bootloader programs in bare metal systems, regardless of various features such as redundancy, security, safety, is shown in Fig. 1

Upon powering on the system, the microcontroller registers are initialized, and the hardware components are configured to ensure that the processor is in an accessible state. Subsequently, the main program of the bootloader checks the current status of the flash memory and proceeds to load the user configuration data. If the system has progressed without encountering any issues thus far, a stage of selection arises, determining whether to switch to boot or application mode. Microcontrollers commonly employ various methods, such as using input-output (I/O) and buttons, for boot mode selection. In this selection process, if the system transitions to boot mode, the necessary procedures for loading a new program are initiated. Conversely, in the case of transitioning to application mode, the system verifies the presence of a valid application software previously loaded into the flash memory and proceeds to execute it by transitioning to the application's designated starting address. Classic bootloader software is not much different from a standard application software. The competence that makes the classic bootloader software special from standard applications is that it shares the flash memory space with another application and can delete and program a new application instead [16].

### 2.3 Wear leveling algorithms

Wear leveling algorithms are widely employed in flash memory systems, including microcontrollers, to distribute write and erase operations evenly across memory cells. This technique helps extend the lifespan of flash memory and prevents premature wear-out of specific cells. The following sections provide a brief overview of commonly used techniques.

#### 2.3.1 No wear leveling

No wear leveling refers to the absence of any specific mechanisms or algorithms to evenly distribute write and erase operations across the flash memory cells. In this approach, the flash memory cells are written and erased without considering their usage history, which can lead to non-uniform wear and early failure of heavily used cells.

#### 2.3.2 Static wear leveling

Static wear leveling is a wear leveling technique that evenly distributes write and erase operations across the flash memory at the time of initialization or during idle periods. It aims to achieve a uniform distribution of usage across all memory blocks by performing block remapping based on wear-leveling tables. Static wear leveling can be implemented using techniques like block swapping or block rotation. Block swapping involves swapping heavily used blocks with less-used blocks to achieve wear leveling, while block rotation rotates the entire block set to distribute the usage evenly [18].

#### 2.3.3 Dynamic wear leveling

Dynamic wear leveling is a commonly used wear leveling technique that redistributes the write and erase operations dynamically across the flash memory. It tracks the usage history of each memory block and dynamically reassigns the data to less-used blocks. This technique aims to achieve a balanced utilization of memory cells, thus prolonging the overall lifespan of the flash memory.

Although dynamic wear leveling does have great improvement on wear leveling, the endurance improvement is stringently constrained by its nature: That is, blocks of cold data are likely to stay intact, regardless of how updates of non-cold data wear out other blocks. In other words, updates and recycling of blocks/pages will only happen to blocks that are free or occupied by non-cold data, where cold data are infrequently updated data [19].

#### 2.3.4 Hybrid wear leveling

Hybrid wear leveling algorithms combine multiple techniques to optimize wear leveling efficiency. The aim is to leverage the strengths of different techniques and optimize wear leveling performance.

Selecting an appropriate wear leveling algorithm depends on factors such as flash memory characteristics, workload patterns, and available resources on the microcontroller. Each algorithm has its own trade-offs in terms of complexity, performance, and overhead.

In our Efficient Bootloader (EB) design, dynamic read and write operations are performed on fixed-length virtual memory blocks at specific addresses. Additionally, due to the absence of garbage collection mechanism and the availability of an adequate number of blocks is checked before writing new data, the occurrence of write amplification [20] effect is not expected.

## 3 Design and implementation

### 3.1 Efficient bootloader design

The efficient bootloader design aims to determine the most suitable flash memory start address according to the size of the data to be written to the flash memory after boot mode. It is desired to have a memory write distribution as homogeneous as possible with the specified addresses. Thus, there will be an effective reduction in flash write times while increasing the durability of the flash memory.

**Figure 2:**
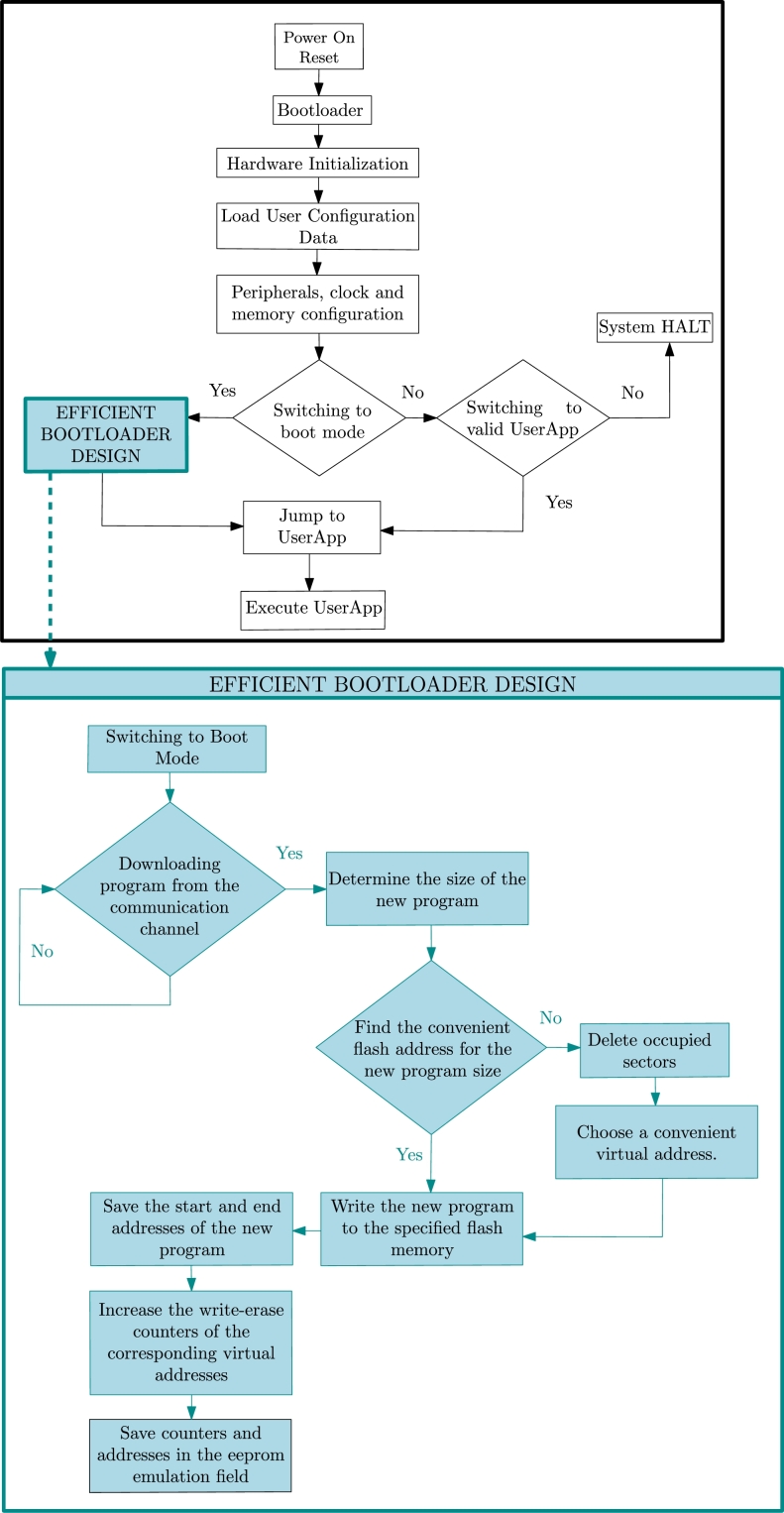
Efficient Bootloader Design.

Efficient and classic bootloader design have basically the same functionality up to boot mode. The efficient bootloader sample design is as shown in Fig. 2.

Unlike classical bootloader designs, the flash memory start address to which the application software will be loaded is dynamically assigned at runtime according to the application size. The dynamic wear leveling algorithm [21] ensures that the application is written to memory during the application loading phase, without exposing the sector to erasure, in cases where there are sequential virtual address blocks of sufficient size.

The proposed efficient bootloader design employs the round robin placement method to address the wear imbalance issue. In this method, instead of starting from the zeroth address, each application program is sequentially written to the next available memory sector, forming a circular arrangement. Once the final memory sector is reached, the placement continues from the beginning of the memory space, thereby achieving a homogeneous distribution of wear across the flash memory.

Notably, during the bootloader loading phase, it is assumed that all sectors undergo an erase operation, establishing a consistent starting point for subsequent application program placements. As a result, no additional erase operation is performed until all sectors are filled or until there are an insufficient number of empty sectors available. This key aspect, where no re-erasing occurs during the programming process until all sectors are filled or until enough empty sectors are unavailable, significantly reduces the overall programming time. This feature stands out as one of the most important factors that enhance the efficiency of this design, as it minimizes the need for repetitive erase operations during the entire process of loading programs.

#### 3.1.1 Efficient bootloader software isolation

Flash memory sectors used instead of virtual blocks in the flash memory area used for design, and these areas should be isolated from the memory areas where applications are written. The objective of this isolation is to safeguard the sector(s) housing the bootloader software, virtual block counters, and virtual block addresses from being affected in the event of application software deletion. This isolation provides several advantages, including the ability to independently develop security, safety, and useful functionalities alongside the application software. Examples of these functionalities encompass access authorization for the bootloader's designated sectors, encryption or decryption of application software, storage of cryptographic keys separate from the application software, as well as the incorporation of additional features into the bootloader software and utilization of diverse communication channels.

#### 3.1.2 Programmable virtual memory blocks

Flash memory sector sizes of microcontrollers may differ from each other, and their flash sectors may not be equal in size [22]. In order to ensure that the bootloader design is generic and efficient, flash memory is divided into virtual blocks independent of sectors. Flash memory sector sizes of microcontrollers may differ from each other, and their flash sectors may not be equal in size [22]. In order to ensure that the bootloader design is generic and efficient, flash memory is divided into virtual blocks independent of sectors. This division is facilitated by a meticulously crafted mapping mechanism that translates virtual addresses to physical addresses, ensuring adaptability across various microcontroller configurations.

At system startup, during the initialization of the bootloader information, the virtual-to-physical address mapping is established. In a predefined static and const array, enumerates the starting addresses of each virtual block within the flash memory. Each entry in this array represents a virtual block, with contiguous addresses within the block and gaps between blocks. The size of each virtual block, set to 16 kB in this implementation, delineates the boundaries for program storage within the flash memory.

When a new program is to be loaded, flash memory sectors will not be deleted if the number of sequential and free addresses in the memory are sufficient in accordance with the size of the program to be loaded in the virtual blocks created in the flash memory. The write operation will continue writing from the next virtual memory start address after each filled virtual block. Each new program load request continues to write without erasing flash memory sectors as long as there are appropriate and sequential memory addresses. After the new programs are loaded into the flash memory, the start and end addresses in the memory are recorded. The Erasure Count (EC) of the virtual memory blocks exposed to the write operation are increased. Thus, counters that showing the durability of all virtual memory block addresses are obtained.

When the virtual blocks in the flash memory do not have enough or sequential memory addresses to load a new program, all sectors that have been written before are deleted and the new program is started to be loaded from the first virtual block address in the first sector. When the program is loaded, the counters of the virtual blocks where the data is written are increased and recorded. After the program loading operation is completed successfully, the processes of jumping to the appropriate address and starting the application have similar processes to the mechanism in the classical bootloader design.

#### 3.1.3 Isolation of virtual memory block information

Efficient bootloader software in flash memory sectors and counter-address information of virtual memory blocks are also isolated. Since the virtual memory block information will be updated after each installation, it is required to be in a different sector so that it does not affect the application and bootloader software. The efficient use of the sector where virtual memory block information is a design issue in itself, as it is an essential factor in the efficiency of other sectors.

In efficient bootloader design, the flash memory area where virtual memory block information is stored consists of at least two sectors. Virtual memory block information is set up in a similar or the same structure as the algorithm of saving the application software in memory, thus eliminating the effects that will reduce the efficiency of the design. The start and end address information and counter information of the virtual flash memory blocks related to the application to be loaded are sequentially recorded in the isolated flash memory sector after each update. When the end of the first flash memory sector reserved for recording is reached, data from the first sector is swapped into the second reserved sector. The first flash memory sector, which is then reserved and occupied, is completely erased. When the end of the second flash memory sector reserved for recording is reached, the data is swapped to the first erased sector and these erase-swap operations continue cyclically.

The use of at least two flash memory sectors ensures that the virtual memory block information is constantly up to date and sector redundancy. One of the important points to pay attention to is that if the sizes of the sectors where the virtual memory block information is kept are not large enough as the size of the recorded data, the sectors that used for recording will quickly fill up and be deleted. The efficiency of the bootloader software will decrease if the number of erasing sectors that containing virtual memory block information is greater than the number of erasing sectors on which applications are loaded. Therefore, the selection of flash memory sectors is critical for each of the bootloader software, virtual memory block information, and application software considerations.

### 3.2 Efficient bootloader software implementation

#### 3.2.1 CLI application

The Command Line Interface (CLI) was written to provide serial communication between the bootloader software and the computer. The scope of the CLI software can be shaped according to the requirements of the design. The CLI software's most essential task is communicating with the bootloader and uploading applications. In our study, CLI software is used for creating executable files apart from essential functions. In addition, in our study, the integrity of the data communication between the CLI and the bootloader is controlled by Cyclic Redundancy Check (CRC) [23].

#### 3.2.2 Generating executable file from user application project

Regardless of the efficient bootloader software, executables must be created by a specific process for user applications to work correctly. Current bootloader design methods generally focus on the process of writing the ready executable file to flash memory. The reason for this is that the executable file is expected to be produced in a way that meets the requirements of the system to be installed by the user. Since the addresses of writing to flash memory are static in classical bootloader designs, a standard way is followed when producing executable files in official example projects offered by vendors to developers [16], [14], [24], [4], [25]. Here, the standard way means that the RAM and FLASH addresses in the linker files are not changed automatically, and the vector offset addresses remain constant unless the user manually changes them. One of the most critical points in this work is that the executable is dynamically obtained from the project files at runtime. The algorithm for creating executable from user application project via CLI-bootloader communication is shown below.

In the Algorithm 1 flow, the file paths of the executable, system and linker files are requested from the user. Then, by calculating the size of the executable file, how much block space will be used in the flash memory is calculated. The required block count is sent to the efficient bootloader and the optimum flash memory start address which specified as “newAddressOffset” is expected from the efficient bootloader. How the efficient bootloader determines the optimum starting address will be explained in the next topic. After the Efficient bootloader determines the optimum address, it sends it to the CLI application. Before proceeding to the next stage, the linker and system files in the project file are backed up.

**Algorithm 1:**
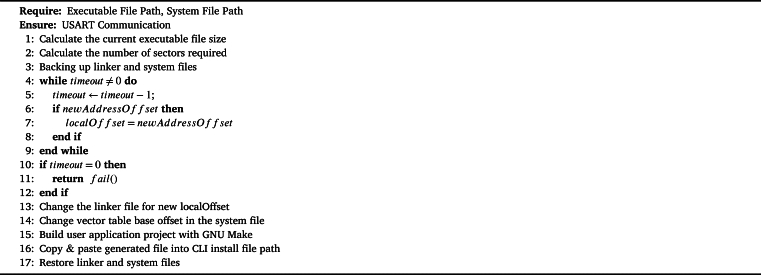
Generate Executable File.

The CLI application, upon receiving the address information, organizes the flash addresses in the linker file. Following the modification of the linker file, the vector table offset values in the project's system file are updated accordingly. Once the CLI application completes the editing of files, it proceeds to compile the project using the GNU's not Unix (GNU) make tool, resulting in the generation of an executable file. A copy of this executable file is then stored in a designated location. Finally, the CLI application concludes the procedure by restoring the modified project files with the previously backed-up files. Subsequently, if the user initiates a flash write command, the CLI application proceeds to load the executable file located in the installation file path into the efficient bootloader. In the event that a write command is issued without prior generation of an executable file, the CLI application will issue a warning message stating, “Executable file is not configured yet. Please create a bin file first.”

In the structure established with the Algorithm 1, the changes that need to be made in the linker file and system files of the new program are handled automatically with the CLI application. Afterwards, the project files were recompiled with the GNU make tool and a new executable file was produced. Thus, the user can generate executable files compatible with the efficient bootloader, without the need for manual configuration to install the new program.

#### 3.2.3 Determining write address for optimal endurance

One of the critical points to achieving optimum flash memory durability is determining the most suitable memory write addresses at runtime. In order to increase the durability of the flash memory, the most suitable flash memory start address is determined at each new program loading stage. Considering the new application size and the free virtual blocks in the flash memory, the memory start address from which the write operation will start is calculated. The calculated flash memory address is then sent to the CLI application. While calculating the flash memory start address, an algorithm similar to the wear leveling algorithm used in the Electrically Erasable Programmable Read-Only Memory (EEPROM) emulation software is used. With this method, it is aimed that all blocks of flash memory allocated to user applications have a homogeneous memory write distribution as much as possible. The most appropriate address determination algorithm for increasing the durability of flash memory is shown in Algorithm 2.

**Algorithm 2:**
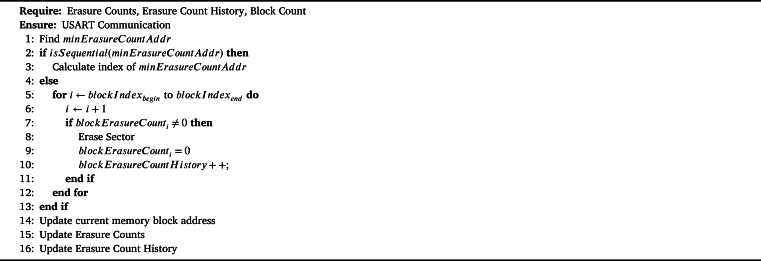
Determining Optimal Address for Endurance.

When a new program load request is received by the Command Line Interface (CLI), the algorithm determines the memory block with the minimum erasure count, denoted as minErasureCountAddr. If the memory blocks from minErasureCountAddr to the end of memory provide enough free space, the algorithm calculates the best index address based on the minErasureCountAddr. However, if there is insufficient free space, all sectors associated with blocks having an erasure count greater than zero are erased.

The blockErasureCount variable represents the current state of the sector, indicating whether it has been erased or not. In the context of the algorithm, if $blockErasureCount_{i}$ is not equal to zero, it means that the sector has been programmed before and needs to be erased.

The blockErasureCountHistory keeps track of the historical erasure count values of each memory block from the initial programming onwards. This information is essential for wear leveling and statistical purposes, as it allows for the analysis of the block's usage and wear patterns over time. The blockErasureCountHistory is not reset after the efficient bootloader design is applied, ensuring that the historical erasure count values are preserved.

After each operation, the algorithm updates the memory block addresses, erasure counts, and erasure count history values. These updated values are stored in dedicated sectors reserved for emulating EEPROM functionality.

## 4 Efficient bootloader performance analysis

In efficient bootloader design, flash memory is divided into 3 partitions. The first of the partitions represents the flash memory sectors where the bootloader software is located, which will change the least compared to the other partitions. The second partition represents the virtual memory blocks of the programs to be loaded into the flash memory. In the third part, the flash memory start address, program size and erasure count of virtual memory blocks are stored.

In order to measure performance, STM32F407VGT6 microcontroller with ARM Cortex-M4 core was used in design and implementation. The STM32F407VGT6 microcontroller has 1 Megabyte of flash memory that can be used to store programs and data [7]. STM32F407VGT6 microcontroller flash memory respectively consists of 12 sectors, 4 sectors x 16 kilobytes, 1 sector x 64 kilobyte and 7 sectors x 128 kilobytes [7]. The flash memory endurance of the STM32F407VGT6 microcontroller is specified as a minimum of 10000 cycles [7]. In efficient bootloader design and implementation, sectors from the fifth to the twelfth sector (Sector 4,5,6,7,8,9,10,11) are used as the program area where user applications will be installed.

### 4.1 Performance of programmable virtual memory blocks

“Sector 4,5,6,7,8,9,10,11” sectors, which are the last 7 sectors of the STM32F407VG microcontroller flash memory, are used to load user applications. The total programmable space is 960 kilobytes, which is divided into 60 virtual memory blocks of 16 kilobytes. In efficient bootloader design, flash memory endurance varies depending on the size of the application software to be loaded.

In the classical bootloader design, we only need to know the endurance capacity to find out how many times we can load a program of a certain size into the flash memory of a microcontroller. Because, in classical bootloader designs, the write start address in the flash memory where a new User Application (UserApp) program will start to be loaded is certain and it will be subject to deletion before each installation. Therefore, the flash memory endurance capacity will decrease with each program load phase.

In the Efficient bootloader design, the new program size directly affects the endurance capacity. Because the efficient bootloader flash memory sectors will not erase as long as there is space in the flash memory sectors to load a new UserApp program. Therefore, the endurance capacity will not decrease until the erasure takes place.

(1) $$PCC_{EB}=\frac{SectorSize\times EnduranceCapacity}{UserAppSize}$$

(2) $$PCC_{CB}=EnduranceCapacity$$

(3) $$EIR=\frac{PCC_{EB}}{PCC_{CB}}$$

Multiplication of total flash memory sectors size and flash memory endurance determines the total memory capacity. By dividing the total memory capacity by the UserApp size, we get the Programming Count Capacity (PCC) value, which shows how many times we can program the microcontroller. The PCC is calculated in Equation (1) for the efficient bootloader and Equation (2) for the classical bootloader. The Endurance Increase Rate (EIR) in the programming capacity of the efficient bootloader design compared to the classical bootloader design is calculated in Equation (3).

Table 1 shows the variation of flash memory endurance for PCC${}_{CB}$ and PCC${}_{EB}$ according to the different sizes of UserApp. Since the first two sectors are reserved for Bootloader software and the next 2 sectors for eeprom emulation software, the available sectors count covers sectors 4 to 11. According to the available sectors count value, UserApp can reach you up to 960 KB.

**Table 1:** STM32F407VGT6 Flash Memory Endurance Efficiency by Boot Scenarios.

Boot Scenarios For Variable Program Sizes						
Endurance Capacity	Available Sectors Count	Available Sectors Size (KB)	UserApp Size (KB)	PCC${}_{CB}$	PCC${}_{EB}$	EIR
10000	Sector 4-11	960	8	10000	1200000	120
10000	Sector 4-11	960	16	10000	600000	60
10000	Sector 4-11	960	32	10000	300000	30
10000	Sector 4-11	960	64	10000	150000	15
10000	Sector 4-11	960	128	10000	75000	7
10000	Sector 4-11	960	960	10000	10000	1

In Table 1, the EIR for a UserApp size of 128KB is shown as 7, rather than 7.5. This distinction arises from the specific arrangement of the application data within the flash memory.

When the UserApp size is 128KB, it is stored in consecutive memory regions comprising one 64KB region and seven 128KB regions. In this configuration, the EIR can only reach a value of 7 due to the nature of consecutive storage. An EIR of 7.5 can only be attained when the application is stored in non-consecutive flash memory regions.

Hence, considering the given scenario where the UserApp is stored in consecutive memory regions, the EIR is correctly reported as 7, indicating the efficiency of the endurance for the STM32F407VGT6 flash memory under these circumstances.

Upon examining Table 1, it is evident that as the size of user application programs increases, the frequency of programming operations performed without sector erasure decreases, leading to a decrease in the gains in endurance capacity.

Flash memory endurance can be increased for cases where the total size of the programs to be loaded is less than the total sector size of 960 kilobytes.

In cases where flash memory sectors have different sizes, the erasing times of sectors differ from each other. The erasing times of the flash sectors of the STM32F407VGT6 microcontroller are shown in Table 2.

**Table 2:** STM32F407VGT6 Flash Memory Sector Erase Durations [7].

Conditions	Sectors	Size (KB)	ET (ms)	DWWT (us)
*TA* = 0 to +40^∘^*C*, *VDD* = .3*V*,*VPP* = 8.5*V*	Sector 0..3	16	230	16
	Sector 4	64	490	16
	Sector 5..11	128	875	16

The Double Word Writing Time (DWWT) of the STM32F407VG microcontroller is equal in all sectors [7]. As shown in Table 2, Erasure Time (ET) is directly proportional to sector size. In other words, the larger the sector size, the longer ET will take. The size of the sectors reserved for bootloader and eeprom emulation software is 64 KB. All remaining sectors are reserved for UserApp.

(4) $$WT_{EB}(us)=\frac{UserAppSize(byte)\times DWWT(us)}{4}$$

(5) $$WT_{CB}(us)=ET(us)+WT_{EB}(us)$$

The Writing Time (WT) represents the elapsed time required for a user application to be written to the flash memory. In the context of the comparison between the efficient bootloader design and classical bootloader designs, the WT is calculated separately using Equation (4) for the efficient bootloader and Equation (5) for the classical bootloader.

Table 3 illustrates the writing time of applications with different sizes to the respective sectors for both the efficient bootloader and classical bootloader designs. The “IM” values indicated in the table denote cases of “Insufficient Memory (IM)”.

**Table 3:** Writing Time For User Applications With Different Sizes.

Sectors	Sector Size (KB)	ET (ms)	DWWT (us)	WT(us)									
				UserAppSize${}_{EB}$(KB)					UserAppSize${}_{CB}$(KB)				
				8	16	32	64	128	8	16	32	64	128
Sector 4	64	490	16	32768	65536	131072	262144	IM⁎	522768	555536	621072	752144	IM⁎
Sector 5-11	128	875	16	32768	65536	131072	262144	524288	907768	940536	1006072	1137144	1399288

⁎ IM: Insufficient Memory

According to Table 3, while only the program writing time is calculated in the efficient bootloader design, the time spent for deleting the sector is added in addition to this time in the classic bootloader design. The times required for applications of different sizes to be loaded once are shown in Table 3.

The efficient and classical bootloader writing time ratio for user applications of different sizes are shown in Table 4. Using the Equation (3), the proportions of programming times between efficient and classical bootloader designs are calculated in Table 4.

**Table 4:** Efficient and Classical Bootloader Writing Time Ratio for User Applications of Different Sizes.

Sectors	WT Efficiency for UserApp of Different Size (KB)				
	8	16	32	64	128
Sector 4	15.95361	8.476807	4.738403	2.869202	IM⁎
Sector 5-11	27.70288	14.35144	7.67572	4.33786	2.66893

⁎ IM: Insufficient Memory

The values shown in the Table 4 represent the ratio of efficient bootloader vs. conventional bootloader memory write times. In the efficient bootloader design, write time increases, and WT efficiency decreases as application size increases. Since the size of Sector 4 is less than the other sectors in the table, WT efficiency is also lower. This is because when the sector size is smaller, classic bootloaders spend less time erasing the sector.

(6) $$EC_{EB}=\lceil \frac{ProgramCount\times UserAppSize(byte)}{AvailableSectorSize(byte)}\rceil$$

(7) TET(ms)=EC×ET(ms)×NumberofSectorUsed

(8) TWT(ms)=(ProgramCount×WT(ms))+TET(ms)

where TET=TotalErasureTime,

EC=ErasureCount,

TWT=TotalWriteTime,

In Equation (6), the total memory requirement is obtained by multiplying the program size to be loaded by the program upload count. The total memory requirement is divided by the sector size to find the erasure count of the sector. The calculation of total spend time to delete sectors is shown in Equation (7). The calculation of the Total Erasure Time (TET) and Total Write Time (TWT) are shown in Equation (8).

The cases where the programs to be loaded are written to all programmable virtual memory blocks are shown in Table 5.

**Table 5:** Efficient And Classic Bootloader Write Time Efficiency For User Applications With Different Sizes.

Available Sectors Count	ET for Sector 4 (ms)	ET for Sector 5-11 (ms)	DWWT (us)	Available Sectors Size (KB)	UserApp Size (KB)	Program Upload Count	EC${}_{EB}$	EC${}_{CB}$	TWT${}_{EB}$ (ms)	TWT${}_{CB}$ using Sector 4 (ms)	TWT${}_{CB}$ using Sectors 5-11 (ms)
Sector 4-11	490	875	16	960	8	10000	84	10000	883340	5227680	9077680
Sector 4-11	490	875	16	960	16	10000	167	10000	1760065	5555360	9405360
Sector 4-11	490	875	16	960	32	10000	334	10000	3520130	6210720	10060720
Sector 4-11	490	875	16	960	64	10000	667	10000	7033645	7521440	11371440
Sector 4-11	490	875	16	960	128	10000	1334	10000	12900040	18892880	13992880
Sector 4-11	490	875	16	960	960	10000	10000	10000	105471600	105471600	IM⁎

⁎ IM: Insufficient Memory

In Table 5, write times are compared for efficient and classical bootloader applications when programs of different sizes are loaded the same number of times. In the same table, the write times of programs with different sizes in the same type of bootloader designs were also compared. According to Table 5, while the sector erasure count is fixed regardless of program size in classical bootloader designs, the sector erasure count increases as the program size increases in efficient bootloader designs. The reason for this is that the free spaces in the flash memory of the microcontroller fill up faster as the size of the program to be loaded increases and the deletion process must be performed in order to be able to rewrite data. The increase in the sector erasure count will cause an increase in the time spent for the deletion process, so the total write-erasure time spent increases. Since the sector erasure time is proportional to the sector size, the case that the program to be loaded does not fit in a single sector is considered as a very inefficient situation in terms of the time spent in classical bootloader designs. As an example of this situation, the write-erasure count obtained when the 128 kilobyte program is written from Sector 4 for the classical bootloader design are shown in Table 5. The program, which has 128 kilobytes in size, starts from Sector 4 (64 kilobytes) and occupies half the space of Sector 5 (128 kilobytes). Since the program to be loaded does not fit in a single sector, it is necessary to delete both sectors in each program load process in classical bootloader designs. This increases the time it takes to load the program. In order to better show the effect of the size of the sector in which the program will be written on the total write time in classical bootloader designs, two different value categories have been created in Table 5. Total write times were calculated by using sector start addresses of 64 kilobytes in the first category and 128 kilobytes in the second category. Since all sectors are tried to be used as equal as possible in efficient bootloader design, such a difference does not occur. If the efficient bootloader and classical bootloader designs have equal sector erasure count, there will be no efficiency difference in erasing and writing times. Efficient bootloader designs, by their nature, cannot have more sector erasure count than classical bootloader designs. Therefore, the efficient bootloader design does not lose any efficiency compared to the classical bootloader design. The greatest possible inefficiency in efficient bootloader design is scenarios where it has the same efficiency as a classic bootloader design.

### 4.2 Predicting flash memory corruption with efficient bootloader

In the memory region where the virtual memory block information of the efficient bootloader design is kept, the P/E number past values are recorded after each update. P/E number historical values keep the number of times virtual memory blocks are written and deleted. By comparing the endurance capacity determined by the manufacturers for the flash memories in the microcontrollers and the past values of the P/E number, it has become possible to predict the durability of the memory. Past values of P/E number directly correspond to durability value. Therefore, by looking at the memory endurance value of the manufacturers, it is possible to warn the users in case the P/E number historical value approaches the limit values. Depending on the application software, memory health checks can be performed at every system startup or runtime. Thus, possible damage or losses can be predicted in advance.

### 4.3 Additional overhead of the algorithm utilized in an efficient bootloader

The efficient bootloader design aims to reduce memory wear by providing a more effective data storage and loading process compared to traditional bootloader approaches. However, along with the advantages offered by this new design, there are additional overheads to consider.

Firstly, the validation of each data packet through CRC during the loading of a new program is a crucial step to ensure reliability. However, as the size of the loaded program increases, this validation process also exhibits a linear increase. Therefore, the extension of CRC validation time for larger programs can impact the overall loading time.

Secondly, unlike classical bootloaders, the efficient bootloader executes an additional control algorithm to dynamically determine the memory address where the new program will be written. This is essential to ensure the homogeneous utilization of memory space.

The time tables forming the basis of the efficient bootloader design have been calculated without taking into account the CRC verification and determination of the memory address where the new program will be written. This approach reflects a process model where writing or erasing operations in memory take place independently of CRC verification and memory address determination processes.

Initially, before loading the new program, CRC verification and memory address determination processes are carried out. At this stage, the integrity of the program is verified, and the target address in memory is identified. However, once these processes are completed, writing or erasing operations in memory are initiated. These operations occur without interacting with any independent process.

One of the key advantages of this design is that the CRC verification and address determination times do not have a direct impact on the writing or erasing times in memory. This allows for a faster new program loading process and homogeneous memory wear. Thus, this design combines high reliability and fast loading performance, offering an effective bootloader solution.

## 5 Conclusions

The write-erase capacities of flash memories in microcontrollers significantly impact the overall durability of the flash memory. During the process of writing data to flash memory, it is possible to program each bit from 1 to 0, while reversing this process from 0 to 1 is not feasible. Flash memory erasure can be performed either at the sector level, where individual sectors are erased, or on a larger scale encompassing multiple sectors. In classical bootloader designs, flash memory durability and flash erasing times during programming are independent of the program's size to be loaded. Considering the studies where microcontrollers are programmed at short intervals, such as experimental studies or the testing phase of industrial products, increasing the endurance of flash memory rather than flash memory retention times has been highlighted. However, the loaded application size directly affects flash memory endurance and programming cycle time with an optimized, efficient bootloader design. In particular, the problems experienced in the production and supply processes of semiconductor-containing products, which started with the pandemic, and the aim of increasing the life span of microcontrollers used in test and experimental processes inspired this research. Since the bootloader software and sample applications offered to users by the current microcontroller manufacturers are configured to start from a fixed flash memory address, erasing cycles made to the same address shortens the lifetime of flash memories. As a result of this study, which was obtained by producing the wear leveling algorithm and its derivatives, the durability of flash memory storage systems was increased without the need for external hardware by optimizing the number of write-erase cycles and the time spent on these processes, with an efficient bootloader design. As a result of the study, in addition to shortening the memory write times, reducing unnecessary memory erase operations, and increasing the durability, the corruption that may occur in the memory due to the durability has become predictable thanks to the efficient bootloader design.

## CRediT authorship contribution statement

**Mehmet Ugur Kebir:** Writing – review & editing, Writing – original draft, Visualization, Validation, Software, Resources, Project administration, Methodology, Formal analysis. **Firat Kacar:** Writing – review & editing, Validation, Supervision, Project administration, Formal analysis, Conceptualization.

## Declaration of Competing Interest

The authors declare that they have no known competing financial interests or personal relationships that could have appeared to influence the work reported in this paper.

## Footnotes

1 https://git.ti.com/cgit/hercules_examples/hercules_examples/tree/Bootloaders/, Jul 2021

2 https://github.com/STMicroelectronics/STM32CubeF4/, Jul 2021
